# Computational Fluid Dynamics Modeling of Rope-Guided Conveyances in Two Typical Kinds of Shaft Layouts

**DOI:** 10.1371/journal.pone.0118268

**Published:** 2015-02-13

**Authors:** Renyuan Wu, Zhencai Zhu, Guohua Cao

**Affiliations:** Department of Mechanical Design and Theory, School of Mechatronic Engineering, China University of Mining and Technology, Xuzhou, Jiangsu, China; China University of Mining and Technology, CHINA

## Abstract

The behavior of rope-guided conveyances is so complicated that the rope-guided hoisting system hasn’t been understood thoroughly so far. In this paper, with user-defined functions loaded, ANSYS FLUENT 14.5 was employed to simulate lateral motion of rope-guided conveyances in two typical kinds of shaft layouts. With rope-guided mine elevator and mine cages taken into account, results show that the lateral aerodynamic buffeting force is much larger than the Coriolis force, and the side aerodynamic force have the same order of magnitude as the Coriolis force. The lateral aerodynamic buffeting forces should also be considered especially when the conveyance moves along the ventilation air direction. The simulation shows that the closer size of the conveyances can weaken the transverse aerodynamic buffeting effect.

## Introduction

Shaft hoisting system plays a significant role in underground mining industry, which is used to transport ore, equipments and personnel and is called “the throat” of shaft. Compared with fixed guide, rope guide is characterized by shorter construction times, lower capital costs, easier maintenance and smoother travelling [1, 2]. Rope guides have been used widely in mining shafts. The behavior of rope-guided conveyances is so complicated that the rope-guided hoisting system hasn’t been understood thoroughly so far.

In order tofigure out the behavior of rope-guided conveyances, researchers made enormous efforts in the past decades. Measuring more than ten mines with laser oscillation finder, Chen [3, 4] found out that displacements of conveyances are inconsistent with the Belyi’s formula [5] calculation results and the Coriolis force had little influence on conveyances. Buchinski [6] accurately obtained 175 discrete skip flight records using an Onboard Strapdown Inertial Navigation System (INS), and the results indicate that the magnitude of conveyance horizontal motion is related to residual unbalanced head/tail rope torque, Coriolis force and hoisting speed. Actually, aerodynamic force which is related to the hoisting speed and ventilation play an important role on the horizontal motion of rope-guided conveyances [2, 7, 8, 9]. With the empirical formula for aerodynamic forces, Greenway [2] simulated the rope-guided hoisting system in Matlab, and pointed out that the aerodynamic coefficient is ideally defined by means of wind tunnel testing or computational fluid dynamics (CFD) analysis. Hamilton [7] gave a brief introduction of CFD to study the rope-guided system. With the CFD analyses, Krige [8] gave simplified equations to calculate the conveyance displacements approximately.

Our previous work presented a fluid-structure interaction (FSI) method to simulate the lateral oscillations of rope-guided conveyances in two-dimension (2D) [10]. However, three-dimensional (3D) simulation was more reasonable. In this study, we used FSI method to simulate rope-guided hoisting system in three-dimension (3D) and to investigate the differences between the rope-guided mine elevator and mine cage.

## Methods

The protocol of the study has been approved by the ethical committee of China University of Mining and Technology. Each subject signed informed consent before recruited in the study.

The detailed fluid-structure interaction (FSI) technique to simulate the lateral oscillations of rope-guided conveyances can be found in our previous article [10].

### Equations of lateral motion

**Fig 1:**
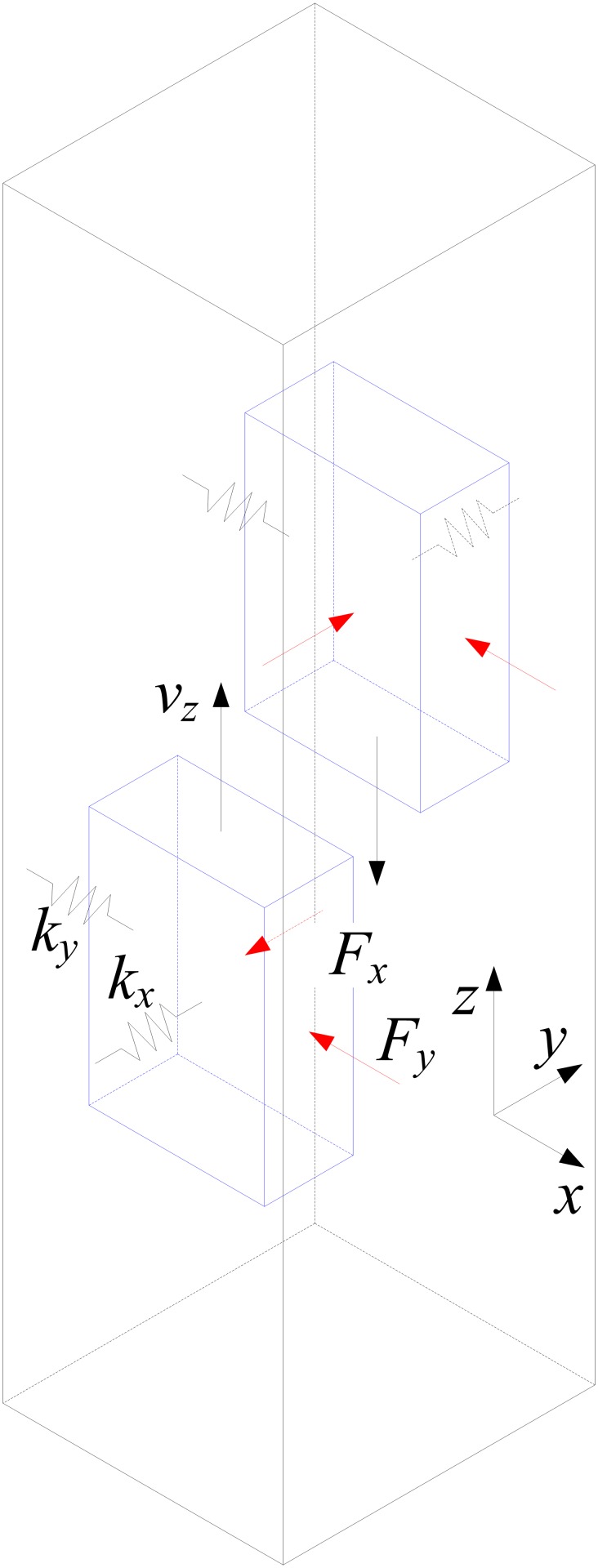
Mathematical model of rope-guided hoisting system.

Fig. 1 gives the mathematical model of rope-guided hoisting system. In some Chinese mine hoisting practices, the thrust bearings are equipped with the hoist rope attachment and the tail rope attachment, which can eliminate the torsion from hoist rope and tail rope. So the rotation of the conveyance about the vertical axis is omitted in this study. The equations of horizontal motion for the conveyance in x-direction and y-direction can be represented as follows [10]:

$$m\ddot{x}+kx=F_{x},$$ (1)

$$m\ddot{y}+ky=F_{y},$$ (2)

where *m* represents the mass of conveyance, x and y represent the displacements of conveyance in x-direction and y-direction respectively, k represents the lateral equivalent spring stiffness for conveyance, and *F*_*x*_ and *F*_*y*_ represent the disturbing forces on conveyance in x-direction and y-direction respectively [2]. The lateral equivalent spring stiffness of rope-guided conveyance can be found in references [8, 10].

Coriolis effect causes westward movement for a conveyance travelling upward and eastward movement for a conveyance, so the disturbing forces in east-west direction consist of aerodynamic forces and the Coriolis force, and in north-south direction exclude the Coriolis force. The magnitude of the Coriolis force *F*_*c*_ can be represented as follows [8]:

$$F_{C}=2mv_{z}\omega \cos\phi ,$$ (3)

where *m* is the mass of conveyance, including mass of rope attachments and payload, *v*_*z*_ is the hoisting speed of conveyance, *ω* is the radial rotation velocity of the earth, and *ϕ* is latitude of the mine shaft site.

### Shaft details

**Fig 2:**
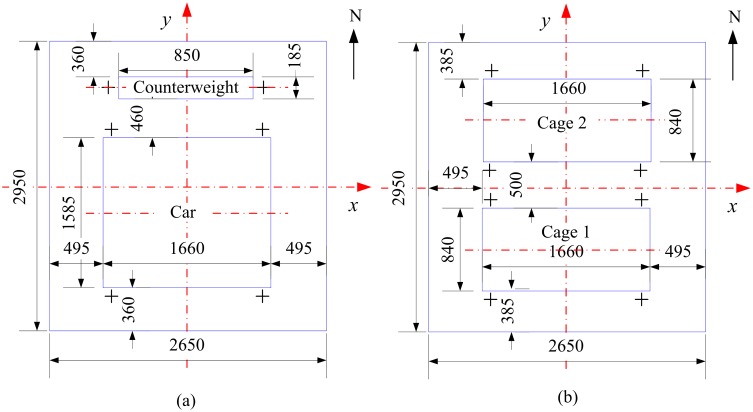
Two typical kinds of shaft layouts. (a) Mine elevator layout and (b) mine cages layout.

**Fig 3:**
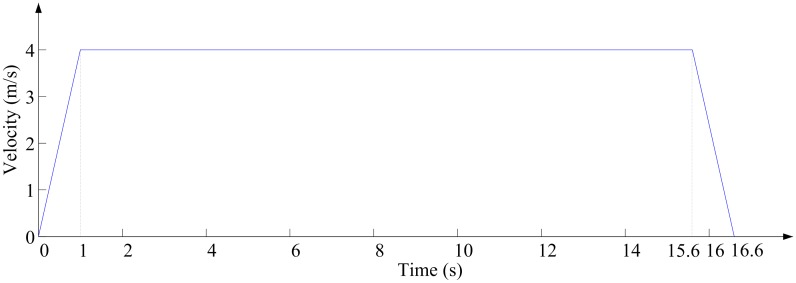
Time-speed diagram in vertical direction.

Fig. 2 gives two typical kinds of shaft layouts: mine elevator shaft layout and mine cage shaft layout, and in this study, the blockage ratios for these two kinds of shaft layouts are same. The main parameters of shaft layouts are given in Table 1, and the time-speed diagram in vertical direction of two kinds of shaft layouts are also same, as Fig. 3 shown.

**Table 1:** The main parameters of shaft layouts.

	Mine Elevator	Mine Cages
Head ropes	4 off, 13 mm, 0.586 kg/m	4 off, 13 mm, 0.586 kg/m
Tail ropes	1 off, 26 mm, 2.33 kg/m	1 off, 26 mm, 2.33 kg/m
Guide ropes	4 off for car, 2 off for countweight, 24 mm, 3.22 kg/m	4 off per cage, 24 mm, 3.22 kg/m
Conveyance self mass	Car 2.1 t, Countweight 2.6 t	1.6 t
Hoisting distance	62.4 m	62.4 m
Hoisting speed	4.0 m/s	4.0 m/s
Conveyance payload	1 t	0.8 t
Method of tensioning	Weight	Weight
Tension load at shaft bottom	1000 kg per guide rope	1000 kg per guide rope
Ventilation air speed	2 m/s downcast	2 m/s downcast

### Mesh characteristics and solver

**Fig 4:**
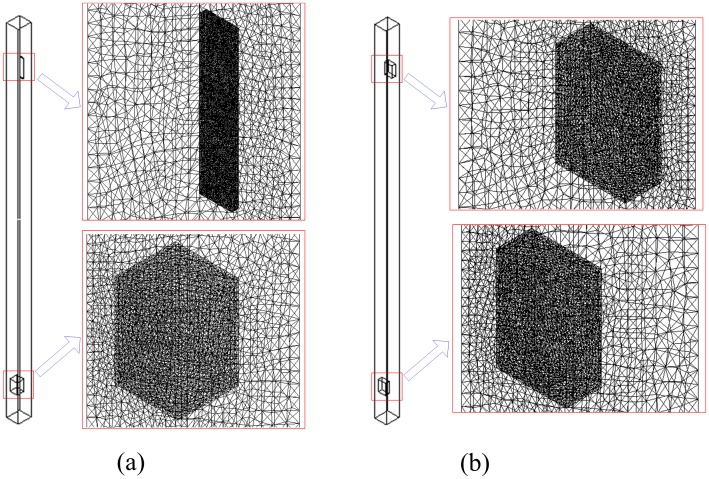
Meshes used for calculations. (a) Mine elevator meshes and (b) mine cages meshes.

The commercial code, ANSYS FLUENT 14.5, has been employed to simulate these two transient cases with PISO scheme. In this study, the ventilation air flow is downcast, so the upper entrance is a velocity inlet boundary condition and the lower exit is pressure outlet boundary condition. All other boundary conditions are no slip at the wall. User-defined functions (UDFs) were written to define the vertical velocity of conveyances and to solve the equations of horizontal motion for conveyances with the Newmark-beta method ($\gamma =\frac{1}{2},\beta =\frac{1}{4}$) [11]. The movements of conveyance have been implemented using a dynamic mesh with the local cell remeshing method in ANSYS FLUENT. As the local cell remeshing method only affects the tetrahedral cell types in the mesh [12], tetrahedral meshes were generated in the domain. Fig. 4 illustrates the front sectional views and local views of unstructured meshes and there are about 4×10^5^ control volumes. The Reynolds number is about 7.6×10^5^, so the shear-stress transport (SST) *k*—*ω* model [13] was used for the turbulent air flow. These two transient cases were resolved using a characteristic time step of 0.002 seconds, and the total time is 16.6 seconds for the hoisting conveyances. There are 8300 time steps in total and 50 iterations for each maximum time step to resolve each case. About 20 h of CPU time in a 4-node HPC cluster were required to complete each numerical simulation.

## Results

**Fig 5:**
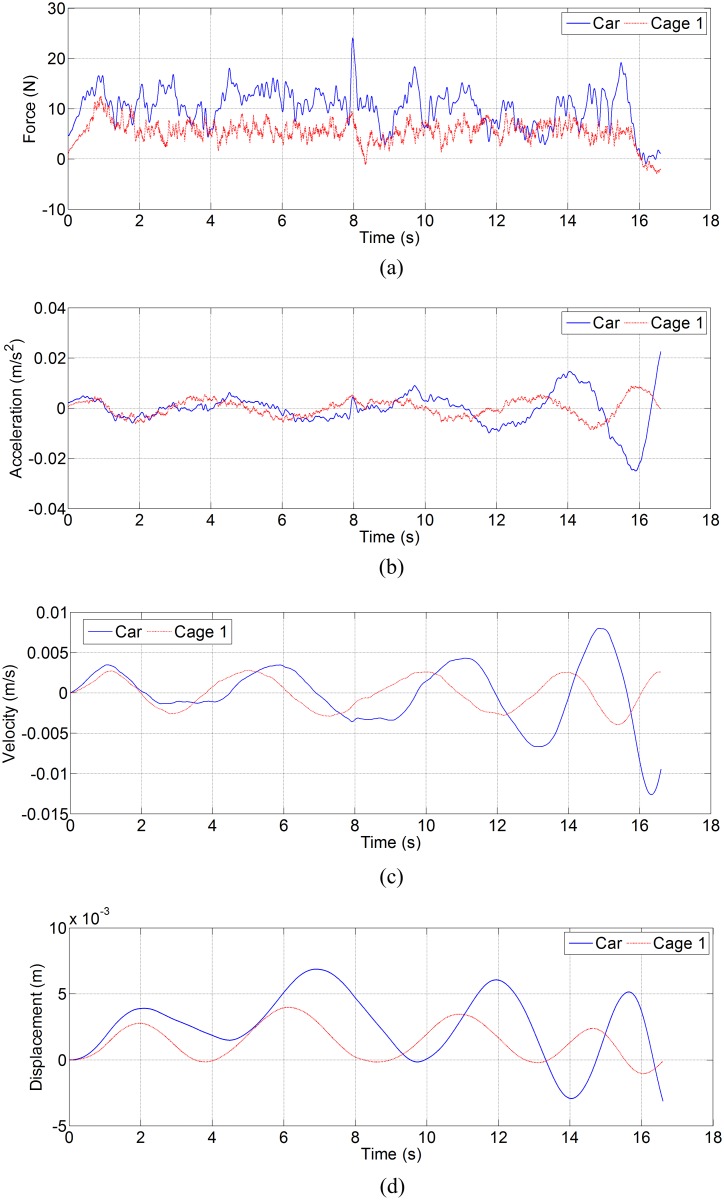
Simulation results for ascending car and cage 1 in north-south direction. (a) Lateral disturbing force, (b) lateral acceleration, (c) lateral velocity, and (d) lateral displacement.

**Fig 6:**
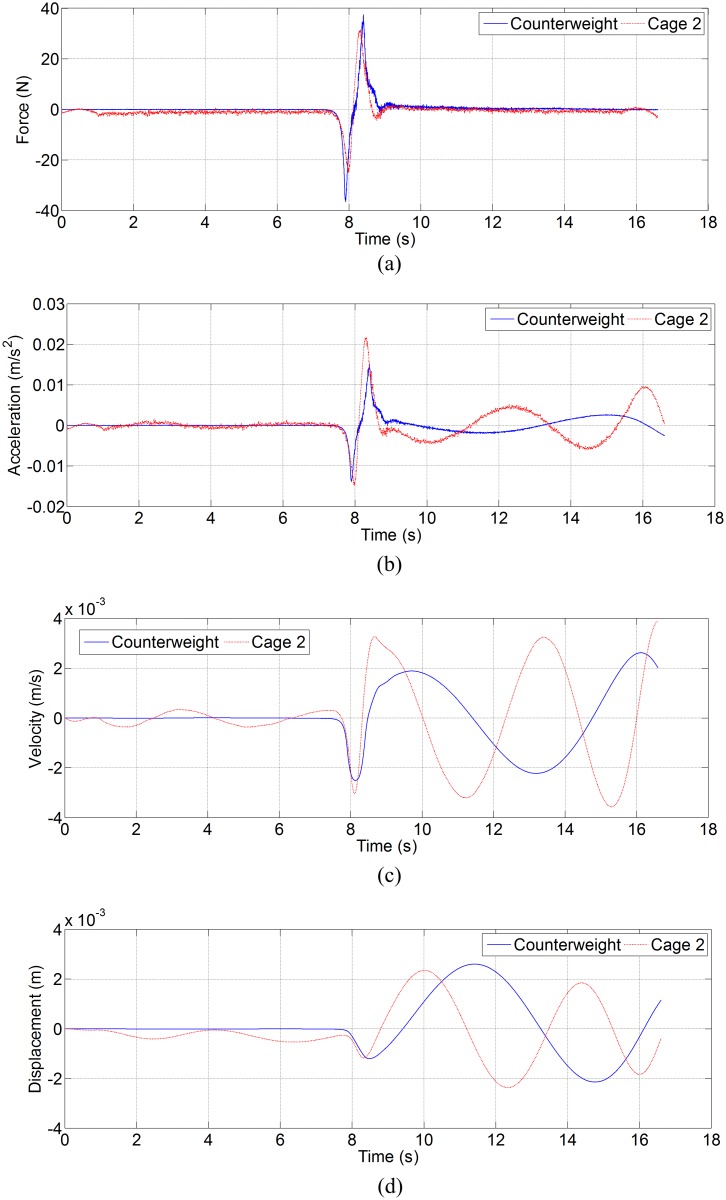
Simulation results for descending counterweight and cage 2 in north-south direction. (a) Lateral disturbing force, (b) lateral acceleration, (c) lateral velocity, and (d) lateral displacement.

**Fig 7:**
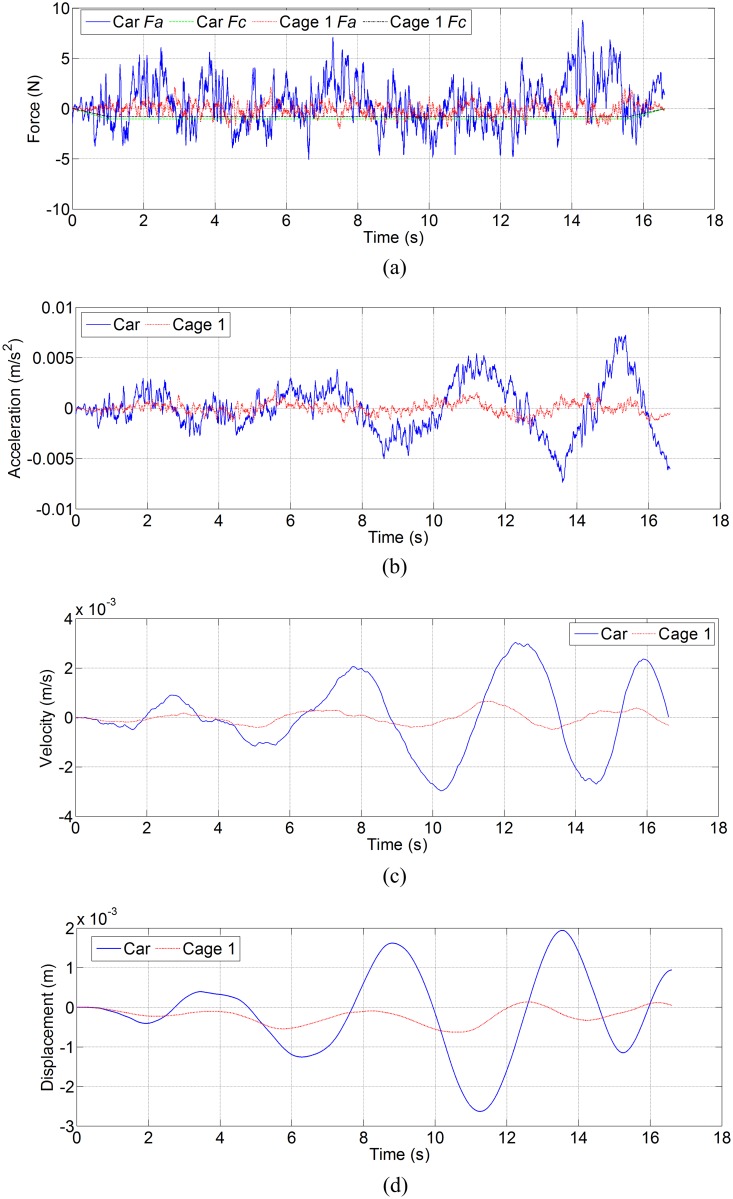
Simulation results for ascending car and cage 1 in east-west direction. (a) Side disturbing force, (b) side acceleration, (c) side velocity, and (d) side displacement.

**Fig 8:**
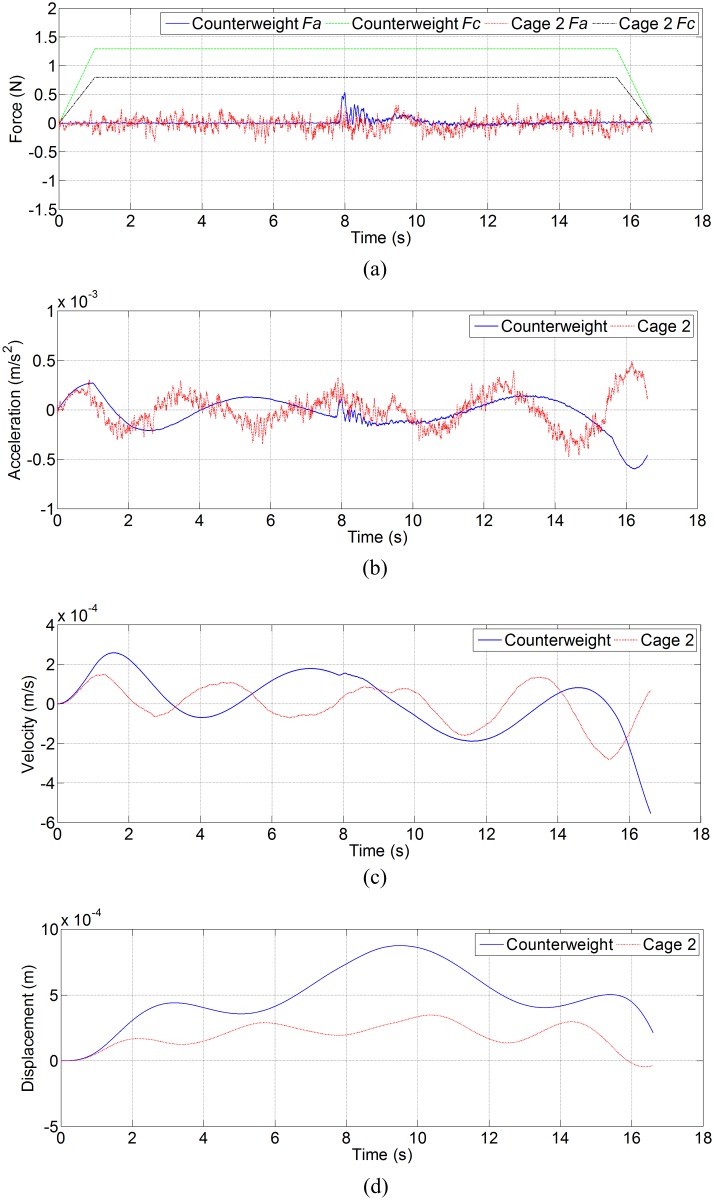
Simulation results for descending counterweight and cage 2 in east-west direction. (a) Side disturbing force, (b) side acceleration, (c) side velocity, and (d) side displacement.

Fig. 5 shows the lateral disturbing force, lateral acceleration, velocity and displacement of ascending car and cage 1 in north-south direction, and Fig. 6 shows the lateral disturbing force, lateral acceleration, velocity and displacement of descending counterweight and cage 2 in north-south direction. Fig. 7 shows the side disturbing force, side acceleration, velocity and displacement of ascending car and cage 1 in east-west direction, and Fig. 8 shows the side disturbing force, side acceleration, velocity and displacement of descending counterweight and cage 2 in east-west direction. The summary of horizontal displacement is shown as Table 2.

**Table 2:** Summary of horizontal displacement.

	Car		Cage 1		Counterweight		Cage 2	
	Lateral	Side	Lateral	Side	Lateral	Side	Lateral	Side
Upper bound (mm)	6.88	1.94	3.99	0.14	2.60	0.88	2.35	0.35
Lower bound (mm)	-3.13	-2.63	-1.03	-0.63	-2.15	0	-2.37	-0.04
Amplitude (mm)	10.01	4.57	5.02	0.77	4.75	0.88	4.72	0.39

## Discussion

As Fig. 5(a) and Fig. 6(a) show, the trend of aerodynamic forces in north-south direction (lateral force) on ascending conveyances are different from those on descending conveyances. Since the ventilation air flows downcast, aerodynamic buffeting forces on descending conveyances are obvious when two conveyances pass each other at mid-shaft. Moreover, aerodynamic buffeting forces also exist for ascending conveyances. The maximum buffeting force on ascending car is about 24 N, that on ascending cage 1 is about 12.5 N, that on descending counterweight is about 37.4 N, and that on descending cage 2 is about 31.4 N. The lateral aerodynamic buffeting force on counterweight is larger than that on car, because the lateral sectional area of counterweight is larger than that of car. As Fig. 7(a) and Fig. 8(a) illustrate, the maximum aerodynamic force in east-west direction (side force) on ascending car is about 8.8 N, that on ascending cage 1 is about 2.1 N, that on descending counterweight is about 0.53 N, and that on descending cage 2 is about 0.35 N. These differences are also mainly caused by the side sectional area, and agree with the empirical formula [2,3,11]. The maximum Coriolis force on car is about 1.0 N, that on cage is about 0.8 N, and that on counterweight is 1.3 N. So the lateral aerodynamic buffeting force in north-south direction is much larger than the Coriolis force, and the side aerodynamic force in east-west directions have the same order of magnitude as the Coriolis force.

As Fig. 6 exhibits, the double-impulse aerodynamic buffeting force gives a double-impulse shock acceleration, and then influences the velocity and displacement of conveyance. This is obvious on descending counterweight and cage 2, because the direction of these conveyances move vertically is as same as the ventilation air, and then the steady aerodynamic force on descending conveyance is much smaller than that on the ascending conveyance, while the aerodynamic buffeting force on descending conveyance caused by the conveyances pass each other is as great as that on the ascending conveyance. The SME mining engineering handbook said that the buffeting forces do not need to be considered when designing a shaft [9], while the simulation shows that the buffeting forces should also be considered especially when the conveyance moves along the ventilation direction.

As Table 2 shows, the lateral displacement of conveyance is much larger than the side displacement, because the lateral force is much large than side force. The lateral and side displacements of car are larger than those of cage 1, and lateral and side displacements of counterweight are also larger than those of cage 2. Given the same blockage ratio, so two uniform cages can better weaken the transverse aerodynamic buffeting effect than the car and counterweight.

## Conclusions

With the UDFs loaded by Fluent, this paper investigates the behabior of rope-guided conveyances in two typical kinds of shaft layouts. Some conclusions are drawn as follow:

1. The lateral aerodynamic buffeting force is much larger than the Coriolis force, and the side aerodynamic force have the same order of magnitude as the Coriolis force.
2. The buffeting forces should also be considered especially when the conveyance moves along the ventilation air direction.
3. The closer size of the conveyances can weaken the transverse aerodynamic buffeting effect.

## Supporting Information

S1 Table Simulation results for ascending car and cage 1 in north-south direction. (XLS)

S2 Table Simulation results for descending counterweight and cage 2 in north-south direction. (XLS)

S3 Table Simulation results for ascending car and cage 1 in east-west direction. (XLS)

S4 Table Simulation results for descending counterweight and cage 2 in east-west direction. (XLS)
